# Effect of Parity and β-Amyloid on Cognition and Hippocampal Volume in Postmenopausal Women

**DOI:** 10.1212/WNL.0000000000218153

**Published:** 2026-06-22

**Authors:** Clara Gallay, Natalia Soldevila-Domenech, David López-Martos, Gonzalo Sanchez-Benávides, Jordi Huguet, Marc Suárez-Calvet, Juan Domingo Gispert, Gemma Salvadó, Erin E. Sundermann, Oriol Grau-Rivera, Anna Brugulat-Serrat, the for ALFA study

**Affiliations:** 1 Barcelonaβeta Brain Research Center (BBRC), Pasqual Maragall Foundation, Spain;; 2 University Pompeu Fabra, Barcelona, Spain;; 3 Hospital del Mar Research Institute, Barcelona, Spain;; 4 Neurology Service, Hospital del Mar, Barcelona, Spain;; 5 Centro de Investigación Biomédica en Red Bioingeniería, Biomateriales y Nanomedicina, (CIBER-BBN), Barcelona, Spain;; 6 Clinical Memory Research Unit, Department of Clinical Sciences Malmö, Lund University, Sweden;; 7 Department of Psychiatry, University of California, San Diego, La Jolla;; 8 Centro de Investigación Biomédica en Red de Fragilidad y Envejecimiento Saludable (CIBERFES), Madrid, Spain;; 9 Global Brain Health Institute, San Francisco, CA;; 10 Faculty of Medicine, University of Vic-Central University of Catalonia (UVic-UCC), Vic/Manresa, Spain; and; 11 Neurology Service, Hospital Universitari General de Vic, CHV, Spain.

## Abstract

**Background and Objectives:**

Epidemiologic studies exploring the effect of parity on Alzheimer disease (AD) yield mixed results, and little is known about how parity may modify the impact of AD pathology on brain and cognitive aging. We examined the effect of parity and AD pathology on hippocampal volume (HV) and cognitive changes in cognitively unimpaired (CU) postmenopausal women.

**Methods:**

Analysis was performed on CU postmenopausal women from the ALFA+ observational cohort based in the BarcelonaBeta Brain research center, who completed 1 or 2 visits spaced by ∼3 years (mean = 3.38, SD = 0.55) with reproductive data, cognitive testing (modified Preclinical Alzheimer's Cognitive Composite), CSF biomarkers (Aβ42 and Aβ40), and structural MRI. We used linear mixed-effects models to investigate the interactive effects of parity, time, and Aβ status (based on CSF Aβ42/Aβ40) on cognition and HV. *APOE*-ε4 status, age, and total intracranial volume were included as covariates.

**Results:**

A total of 254 women were included in our analyses ranging from 49.2 to 73.4 years (mean age = 61.2) at visit 1. A significant interaction between parity and Aβ status was observed for cognitive decline and HV. In women with positive amyloid status, higher parity was associated with steeper cognitive decline (β = −0.035, 95% CI −0.068 to −0.003, *p* = 0.033) and lower HV across time points (β = −0.134, 95% CI −0.263 to −0.005, *p* = 0.036).

**Discussion:**

In CU postmenopausal women, higher parity interacts with AD pathology to influence cognitive decline and HV reductions. These findings suggest that parity may affect resilience to AD pathology in preclinical AD stages.

## Introduction

Alzheimer disease (AD) is a neurodegenerative disorder characterized by the accumulation of pathophysiologic abnormalities leading to neurodegeneration and progressive cognitive and functional decline. β-amyloid (Aβ) protein accumulation, a hallmark neuropathologic marker of AD, is thought to trigger a pathologic cascade that leads to further accumulation of tau protein, neurodegeneration, and cognitive impairment.^1^ This pathologic process particularly affects the hippocampus early in the disease course, making hippocampal volume (HV) a hallmark predictor of cognitive decline in AD.^2^ Being biologically female is one of the main nonmodifiable risk factors for AD.^3^ Women who develop AD show a worse cognitive trajectory after mild cognitive impairment (MCI) diagnosis,^4^ a faster atrophy rate in the hippocampus,^5^ and may benefit less from amyloid antibody-based therapies.^6^ These differences in pathology suggest that sex-dependent pathways or biological processes may drive distinct pathologic trajectories. In particular, pregnancy is a female-specific event whose long-term effect on the brain remains poorly understood.

Pregnancy is a complex biological process which provokes widespread changes to hormonal,^7^ immune,^8^ and metabolic^9^ systems of the body. In addition, significant changes to hippocampal structure and volume have been observed.^10^ Animal models show decreased neurogenesis in the hippocampus during gestation and the postpartum period.^11,12^ While clinical evidence suggests that gestation negatively affects HV up to at least 6 years postpartum,^13^ whether this effect persists in older adults is unknown. These potentially long-lasting structural alterations may be particularly consequential in the context of early AD pathogenesis, in which HV is also affected.^14^ Therefore, parity-induced alterations to the hippocampus may create a neurobiological context that alters the extent to which AD pathology affects cognitive trajectories and HV in later life.

The effect of parity on AD risk and cognitive decline remains poorly understood, and existing evidence paints a complex picture. Some epidemiologic studies suggest a potential association between grand multiparity (i.e., having 4+ children) and increased risk of AD-related cognitive impairment.^15,16^ However, others have shown no association,^17^ an inverse association,^18^ or a nonlinear effect, suggesting that, in addition to grand multiparity, nulliparity might also be associated with worse AD-related outcomes.^19^ Recent evidence indicates that parity modulates women's cognitive performance differently depending on their disease status. Specifically, a recent cross-sectional study of 446 women (207 cognitively unimpaired [CU], 155 with MCI, and 84 with clinically diagnosed dementia) found that higher parity was associated with enhanced executive function but reduced episodic memory only among women with dementia, not among cognitively healthy women.^20^

However, these studies did not include AD-specific biomarkers, leaving the disease etiology uncertain and preventing examination of how parity may influence cognitive and structural trajectories earlier in the AD trajectory. To the best of our knowledge, only 2 studies have accounted for AD biomarkers or neuropathologic changes, with mixed results. A postmortem histopathologic study of 42 women spanning the spectrum from normal aging to definite AD, categorized using the CERAD neuropathologic battery, found that multiparity was associated with a greater number of neuritic plaques.^21^ However, analysis of Aβ-PET data from 248 women, including those with CU or clinically diagnosed MCI, found no association between Aβ burden and parity or multiparity.^22^ These inconsistent findings highlight the need for a more nuanced examination of the interplay between parity and AD pathology in women in the AD preclinical stage. Specifically, it remains uncertain whether parity interacts with Aβ pathology to affect other brain or cognitive outcomes.

To elucidate the aforementioned landscape of mixed results, we investigated the interplay between parity, Aβ status, cognition, and HV in postmenopausal CU women. In particular, we aimed to test whether parity interacts with Aβ load in its association with cognitive decline and HV. We hypothesized that parity would interact with Aβ load in its association with HV and cognitive decline.

## Methods

### Study Participants

We included postmenopausal women who completed the baseline and first longitudinal visit (with an average time between visits of 3.32 years) from the ongoing ALFA+ study (ClinicalTrials.gov, NCT01835717). ALFA+ is a research cohort of middle-aged CU participants, many of whom are offspring of patients with AD, who have been deeply characterized by clinical interviews, lifestyle and risk factor questionnaires, cognitive testing, CSF biomarkers, and neuroimaging procedures, including MRI and Aβ PET. All these procedures are repeated every 3 years with the main aim of identifying the earliest pathophysiologic changes in the preclinical AD continuum.^23^ In brief, ALFA+ inclusion criteria were: (1) participants who had previously participated in the 45–65/FPM2012 study (ALFA parent cohort); (2) age between 45 and 75 years at the moment of inclusion in the cohort; and (3) long-term commitment to undergo all tests and study procedures (MRI, PET, and lumbar puncture). Exclusion criteria at baseline included: (1) cognitive impairment (Clinical Dementia Rating >0, Mini-Mental State Examination <26, semantic fluency <12); (2) any significant systemic illness or unstable medical condition which could lead to difficulty complying with the protocol; (3) any contraindication to any test or procedure; and (4) family history of monogenic AD. In addition, women with a history of cardiovascular-related gestational disease, in this case preeclampsia or gestational diabetes, were excluded from our sample for analysis. Sex and menopausal status, defined as absence of menstruation for 12 consecutive months, were self-disclosed. Women who reported being premenopausal or perimenopausal were not included in our analysis either.

### Reproductive History Variables

Parity was defined as the number of biological children and was treated as a continuous variable in the primary analyses. In sensitivity analyses, parity was either used as a 2-level [nulliparous vs parous] or 3-level [nulliparous (0 biological child), primiparous (1 biological child), or multiparous (≥2 biological children)] categorical variable. This information was self-reported by individuals during an interview with a clinician at the baseline visit.

### Cognitive Measures

The main cognitive outcome of this study was a modified Preclinical Alzheimer's Cognitive Composite (mPACC), including the total immediate recall of the Free and Cued Selective Reminding Test, the total delayed recall of the logical memory subtest in the Wechsler Memory Scale-IV, the Coding subtest of the Wechsler Adult Intelligence Scale-Fourth Edition, and the total score of the semantic fluency test (animals category), as defined in previous work.^24^ *Z*-scores were computed for visits 1 and 2 and used in the linear regressions. When using the longitudinal mPACC score in the mediation analysis, longitudinal cognitive change was computed as the difference between baseline and follow-up standardized scores.

### AD Biomarkers

CSF collection, processing, and storage in the ALFA+ study have been described previously.^25^ In brief, CSF Aβ42 and Aβ40 were measured with the exploratory Roche NeuroToolKit immunoassays (Roche Diagnostics International Ltd., Rotkreuz, Switzerland) on a cobas e 601 module. Measurements were performed at the Clinical Neurochemistry Laboratory, Sahlgrenska University Hospital, Molndal, Sweden. Aβ status (Aβ+, Aβ−) was defined using the cutoff of 0.071 for the ratio Aβ42/40,^25^ where any participant below this cutoff was considered to be Aβ+. This approach reflects the well-characterized threshold effect in amyloid accumulation, where pathologic cascades are triggered beyond a critical level.

### Imaging Data Acquisition and Preprocessing

Information on MRI data acquisition is available in the supplementary material (eMethods 1).

Automatic Segmentation of Hippocampal Subfields software^26^ was used on the T1, T2, and inversion recovery weighted images to segment the hippocampal formation in the following subregions: Brodmann areas 35 and 36; cornu ammonis 1, 2, and 3; dentate gyrus; entorhinal cortex; parahippocampal cortex; subiculum; and the hippocampal sulcus. All segmentations were visually inspected before proceeding with the statistical analyses.

Hippocampal values are reported in cm^3^ and were obtained at both baseline and follow-up visits.

Total intracranial volume (TIV) was estimated using deformation fields obtained during the nonlinear registration of each subject's T1-weighted MRI to a whole-brain template. The FSL Brain Extraction Tool^27^ was applied to the template to create an intracranial mask, which was warped into each subject's MRI at native space to obtain the TIV measurement. TIV is also reported in cm^3^.

### Statistical Analyses

Linear mixed-effects models were used to explore associations between variables through time. This method was chosen over using simple linear regression and using subject slopes between baseline and follow-up due to its ability to account for within-subject correlation, and accommodate missing data under MAR assumptions.

Cognitive performance was designated as the primary outcome of interest, given its direct clinical relevance. HV was examined as a secondary, mechanistic outcome to provide neuroanatomical context for the cognitive findings. Using baseline data, we first examined the association between parity (exposure) and Aβ status (outcome) covarying for age at beseline visit, *APOE*-ε4 carrier status, and years of education.

Our primary analysis assessed the joint effects of parity and Aβ status (exposures) on cognition (outcome) across both visits and tested whether this association depended on time. This model included a 3-way interaction term among parity, Aβ positivity, and time (the interval between visit 1 and visit 2, in years), as well as all lower-order interactions. A hierarchical approach was used to test interactions: when 3-way interactions (parity × Aβ status × time) were significant, they were interpreted as the primary finding, indicating that the relationship between parity and rate of change differed by amyloid status. When 3-way interactions were not significant, we examined lower-order 2-way interactions (parity × Aβ status) to test whether cross-sectional relationships between parity and outcomes varied by amyloid status, independent of longitudinal change. Analyses were adjusted for *APOE*-ε4 carrier status, years of education, and age at the baseline visit.

Our secondary analysis focused on assessing the joint effects of parity and Aβ status (exposures) on HV (outcome) across both visits and to test whether this association depends on time. The same hierarchical approach was used to interpret interaction effects sizes as for the primary analysis. Analyses were adjusted for *APOE*-ε4 carrier status, years of education, age at the baseline visit, and TIV.

For each regression output showing a significant interaction term, sensitivity analyses were performed using parity as a categorical variable to examine whether the effect of having a biological child differed depending on whether the individual had already had a child. For all parity groups, we conducted post hoc comparisons using estimated marginal means. Specifically, pairwise comparisons between parity groups were examined separately within each Aβ status category, with *p*-values adjusted for multiple comparisons using the Tukey method.

Additional linear regressions were performed as exploratory analyses to explore the interaction between parity and Aβ positivity on individual hippocampal subfield volumes using baseline data. Given the exploratory nature of these analyses, the results are reported without correction for multiple comparisons.

Exploratory mediation analyses were also performed to test whether HV mediates the association between Aβ status and mPACC by parity. We proceeded with the mediation model in whole group and then multiparous women only, which consisted of 3 pathways: (1) the effect of baseline Aβ status on baseline HV (path a), (2) the effect of baseline HV on mPACC difference while controlling for Aβ status (path b), and (3) the direct effect of baseline Aβ status on mPACC difference after accounting for the mediator (path c′). The indirect effect was calculated as the product of paths a and b. Statistical significance of the indirect effect was assessed using bootstrap CIs with 5,000 iterations, and the seed was set to 123. Covariates included age at baseline, *APOE*-ε4 carrier status, education, time, and TIV. TIV and HV were scaled.

Proportions of missing data in exposures (parity, Aβ) or outcome variables (mPACC, HV) at each time point are reported (Table 1).

**Table 1:** Descriptive Characteristics of the Sample

Variables	N	No. of biological children						
		Overall (N = 254)	0 (N = 39)	1 (N = 54)	2 (N = 126)	3 (N = 27)	4+ (N = 8)	*p* Value^a^
Age at first visit, mean (SD)	254	60.8 (4.9)	58.8 (4.5)	59.7 (4.9)	61.5 (4.8)	61.7 (4.8)	65.2 (4.2)	<0.001
Education, y, mean (SD)	254	13.0 (3.6)	14.6 (3.7)	12.7 (3.2)	12.6 (3.6)	13.6 (3.8)	10.9 (3.1)	0.013
Aβ positive, count (%)	229	77 (34%)	10 (30%)	15 (31%)	40 (36%)	8 (30%)	4 (50%)	0.8
*APOE*-ε4 carriers, count (%)	254	130 (51%)	19 (49%)	23 (43%)	72 (57%)	13 (48%)	3 (38%)	0.4
Baseline mPACC *z*-score, mean (SD)	234	−0.03 (0.72)	0.18 (0.79)	−0.04 (0.67)	−0.10 (0.68)	0.04 (0.89)	−0.02 (0.73)	0.2
Follow-up mPACC *z*-score, mean (SD)	203	0.02 (0.78)	0.19 (0.82)	−0.08 (0.88)	−0.02 (0.68)	0.28 (0.80)	−0.57 (0.96)	0.050
Baseline HV, cm^3^, mean (SD)	221	5.37 (0.53)	5.36 (0.50)	5.26 (0.53)	5.42 (0.51)	5.45 (0.52)	4.98 (0.79)	0.4
Follow-up HV, cm^3^, mean (SD)	203	5.22 (0.53)	5.21 (0.50)	5.14 (0.50)	5.27 (0.56)	5.24 (0.56)	5.08 (0.36)	0.7
Baseline TIV, cm^3^, mean (SD)	221	1,250 (95)	1,255 (85)	1,222 (93)	1,259 (96)	1,257 (106)	1,251 (85)	0.3

Abbreviations: Aβ = β-amyloid; HV = hippocampal volume; mPACC = modified Preclinical Alzheimer's Cognitive Composite; TIV = total intracranial volume.

a Kruskal-Wallis rank-sum test; Fisher exact test.

Statistical significance was set at *p* < 0.05. Analyses were conducted using R software, version 4.5.1 (The R Foundation for Statistical Computing, Vienna, Austria). The package “emmeans” was used to calculate the estimated marginal means for direct group comparison of predicted effects.^28^ The package tidiverse was used for data processing and plotting.^29^ The gtsummary package was used to format tables.^30^ The package ggeffects was used to extract predicted values from regression models.^31^ The lme4 package was used to fit mixed-effect linear regression models.^32^

### Standard Protocol Approvals, Registrations, and Participant Consents

The ALFA+ study (ALFA-FPM-0311) was approved by the Independent Ethics Committee of Parc de Salut Mar, Barcelona, and is registered on ClinicalTrials.gov on June 30, 2015 (NCT02485730). All participants provided written informed consent, as approved by the Ethics Committee. The study was performed in accordance with the principles of the Declaration of Helsinki. Baseline visits took place between 2016 and 2019, and the follow-up visit between 2019 and 2022.

### Data Availability

The data supporting the findings of this study may be available on a reasonable request from the ALFA study management team.

## Results

### Sample Characteristics

The sample consisted of 254 participants at the baseline visit and 211 participants at follow-up (Table 1). Of the 43 participants who dropped out of the study between visits, 30 withdrew for personal reasons, 3 became unresponsive, 2 died, 5 showed incidental l findings on their structural MRI, and 3 were excluded due to MCI clinical diagnosis. A descriptive table of participants characteristics comparing those who have not dropped out and those who have can be found in the supplementary material (eTable 1).

As presented in Table 1, 39 (15.3%) participants had no biological child, 54 (21.2%) had 1 biological child, 126 (49.6%) had 2, 27 (10.6%) had 3, and only 8 (3%) had 4 or more. Age significantly differed depending on parity. Average follow-up mPACC score significantly differed by the number of biological children. No differences were observed among groups for Aβ status, *APOE*-ε4 carrier status, or HV.

### Interplay Between Parity, Aβ Status, and Cognition

**Figure 1:**
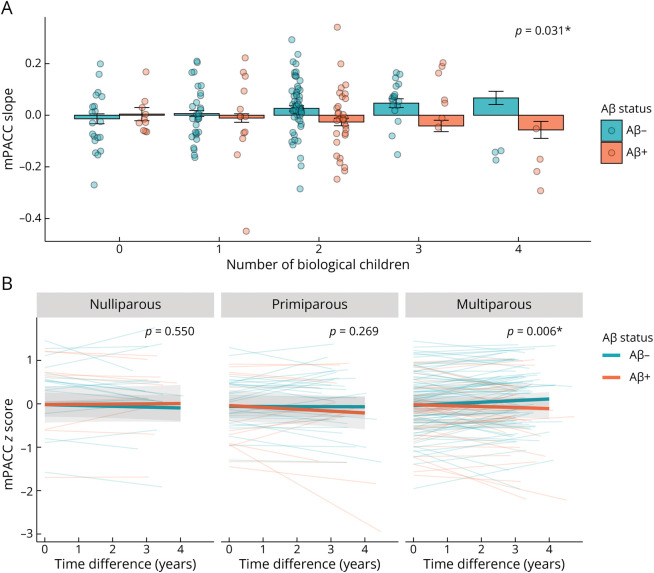
Effect of Parity on mPACC *Z*-Score Change Depending on Aβ Status (A) Estimated mPACC score slope by number of biological children and stratified by Aβ status, over 1 year. mPACC scale ranges from −4 to 4. Error bars represent 95% CIs. Significant value refers to the 3-way interaction outcome in the longitudinal model: parity × Aβ status × time. (B) Predicted values of mPACC change stratified by parity group (nulliparous [0 biological child], primiparous [1 biological child], and multiparous [2+ biological children]) and Aβ status. Lines represent predicted change. Shaded areas represent 95% CIs. Significant values indicate group differences in the predicted slopes, as estimated from marginal means. Aβ = β-amyloid; mPACC = modified Preclinical Alzheimer's Cognitive Composite.

Using baseline data, parity was not associated with Aβ status (p=0.849) (eTable 2). Using longitudinal data (eTable 3), the main effect of parity did not significantly relate to mPACC changes (*p* = 0.614). The 3-way interaction between parity, time, and Aβ status revealed a significant effect on mPACC *z*-score change (β = −0.035, 95% CI −0.068 to −0.003, *p* = 0.031), indicating that Aβ− women showed less cognitive decline with higher parity, whereas Aβ+ women showed steeper cognitive decline with higher parity (Figure 1).

Reproducing the analysis across categorical parity groups (nulliparous, primiparous, multiparous) showed no statistically significant estimate (eTable 4). Group contrast indicated that the difference between Aβ− and Aβ+ participants' predicted cognitive time slope was significant only in multiparous women (β = 0.060, 95% CI 0.017–0.103, *p* = 0.006) (Figure 1). Additional analyses using parity as a dichotomous variable (nulliparous vs parous) are available in the supplementary material (eAppendix 1).

### Interplay Between Parity, Aβ Status, and HV

**Figure 2:**
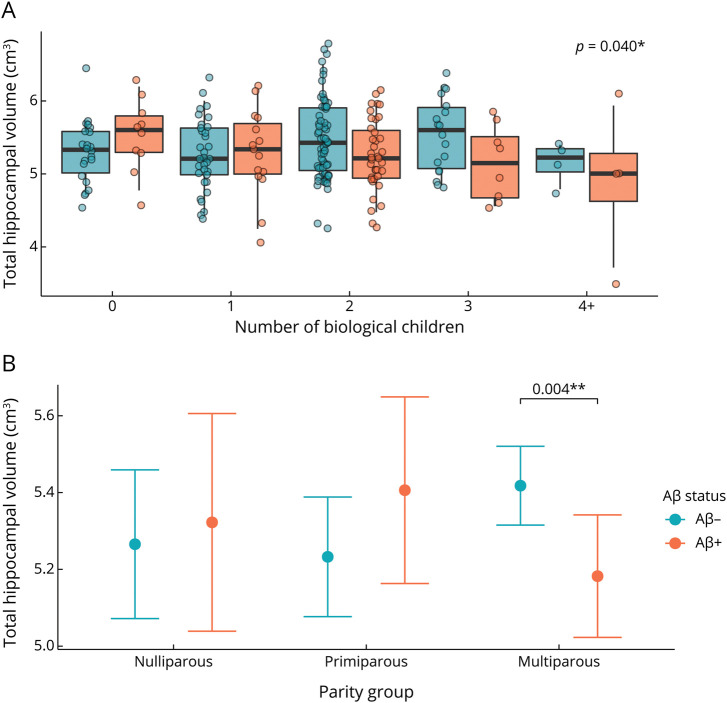
Effect of Parity on Whole HV by Aβ Status (A) Raw HV data at baseline plotted by number of biological children and Aβ status. Whiskers represent 95% CIs. Significant value refers to the 2-way cross-sectional outcome of parity × Aβ status. (B) Predicted values of HV by parity group (nulliparous [0 biological child], primiparous [1 biological child], and multiparous [2+ biological children]) and Aβ load. Error bars represent 95% CIs. Significance values refer to the group contrast of the predicted means using marginal effects at the means. Aβ = β-amyloid; HV = hippocampal volume.

No significant association between parity and HV was found (*p* = 0.206) (eTable 5). The 3-way interaction between parity, time, and HV was not significant either (*p* = 0.166). However, the lower-order interaction between parity and Aβ status was significant (β = −0.134, 95% CI −0.263 to −0.005, *p* = 0.040), showing that higher parity in Aβ+ women was associated with a lower volume (Figure 2). Further exploration of this interaction across hippocampal subfields is available in the supplementary material (eAppendix 2).

When using categorical parous groups as predictors, the resulting model did not yield significant group estimates (eTable 6). However, group-contrast of estimated means indicated that the difference in predicted HV between Aβ− and Aβ+ participants was significant only in multiparous women (β = 0.236, 95% CI 0.074–0.397, *p* = 0.004) (Figure 2). Additional analyses using parity as a dichotomous variable (nulliparous vs parous) are available in the supplementary material (eAppendix 3).

Exploratory mediation analyses examine whether HV mediated the relationship between amyloid and cognition depending on parity groups can be found in the supplementary material (eAppendix 4).

## Discussion

In this study, we examined the associations between parity and cognitive performance and HV and whether Aβ status modified these associations. Parity did not significantly predict Aβ status in our sample nor did it show independent main effects on cognition or HV. Instead, parity interacted with amyloid status to affect both cognitive changes and HV across time points, indicating that reproductive history's influence is contingent on underlying AD pathology. In Aβ-positive women, higher parity was linked with steeper cognitive decline but not in Aβ-negative women. Regarding HV, higher parity was associated with smaller volumes in Aβ-positive women but not in Aβ-negative women. Subgroup analyses indicated that the effect of the parity × Aβ status interaction may be driven by multiparous women. Together, these findings highlight a potential long-term influence of previous pregnancies on AD-related processes in postmenopausal women, suggesting that parity interacts with Aβ pathology to shape cognitive changes.

An interesting result of our study was that parity did not independently affect cognitive changes, but instead, showed an interaction with Aβ status. Our results suggest that the presence and direction of parity's association with cognition may depend on the presence of AD pathologic burden and evoke a deleterious association between higher parity and cognitive change only in the context of Aβ positivity. The observed effect size was small, consistent with the short average time between visits and their baseline clinical status. Still, the comparison of estimated cognitive values between Aβ-negative and Aβ-positive groups was only significantly different in multiparous women, suggesting that pregnancy may influence cognitive change in a cumulative manner. This result offers an explanation for the mixed findings from previous studies examining the relationship between parity and cognitive decline, where sample composition and the lack of biomarker data might have obscured this interaction.^21,22^

Similarly, parity did not predict HV independently but showed an interaction with amyloid load. This suggests that the pathologic burden of amyloid accumulation in HV was associated with reproductive history. Although HV is affected during pregnancy in first-time mothers,^13^ the long-term consequences of this neuronal plasticity remain poorly understood. Previous studies showed that grand multiparity was associated with lower HV in postmenopausal women, but in a sample that included MCI women, who therefore might already have accumulated amyloid pathology.^22^ In healthy rats, parity prevented the age-related decline in hippocampal neural stem cells,^33^ suggesting that similar mechanisms may explain the observed direction of effect in Aβ-negative women. Interestingly, the effects observed in our analyses were independent of time, suggesting that the factors underlying these differences may have occurred before our observation window, which excludes the menopausal transition. Some of these results can be driven by the menopausal transition, given the hippocampus's sensitivity to sex hormones and the importance of this transition for AD onset.^34^ Future research exploring the menopausal transition and its relationship with HV is needed.

There are several additional ways through which parity might interact with Aβ status. First, immune changes induced by pregnancy could drive this interaction. Parity has long-lasting effects on the immune system, as evidenced by the presence of fetal cells in the mother's tissue postpartum.^8^ A key theory behind sex differences in AD trajectory concerns immune differences and their effects on the inflammatory cascade that characterizes Aβ accumulation.^35^ Gestational-related immune changes could contribute to the observed sex differences in immunologic pathways in AD, perhaps through microglial effects on Aβ accumulation. Second, pregnancy's effect on the cardiovascular system could modify later-life cardiovascular risk, which is highly linked with all-cause dementia and AD risk.^36^ Pregnancy requires substantial vascular changes to support the well-being of the new fetus, leading to significant increases in plasma volume and cardiac output.^37^ Previous parity could alter participants' vascular system, which in turn could modulate the effect of Aβ on vasculature, resilience to Aβ accumulation, and AD risk and trajectory. Notably, pregnancy-related vascular complications such as preeclampsia, characterized by endothelial dysfunction, inflammation, and long-term cardiovascular sequelae, may further modify these associations. Given that microglial activation is strongly associated with cerebrovascular injury and Aβ accumulation, vascular and immune pathways may interact. Finally, parity might interact with Aβ status through changes in insulin resistance: higher parity is associated with a higher prepregnancy BMI,^38^ meaning that individuals with higher parity might be more likely to experience insulin-resistance.^39^ Importantly, insulin influences amyloid β peptide clearance, meaning that insulin dysregulation could contribute to its accumulation.^40^

Interestingly, our results may suggest a group difference between primiparous and multiparous women, suggesting that biological and lifestyle factors may operate differently across reproductive histories. Most human studies investigating the effects of pregnancy on brain and hippocampal structure have focused on first-time mothers,^13,41,42^ leaving open the question of whether pregnancy-related changes accumulate with each biological child or occur primarily during the first pregnancy. However, beyond the biological processes associated with pregnancy, it is also essential to consider the transition to motherhood itself, often referred to as matrescence, which involves substantial psychological, social, and lifestyle adjustments.^43^ These experiences, encompassing caregiving demands, stress exposure, and shifts in social roles and routines, may influence brain health through mechanisms distinct from those of pregnancy-related biology. Participants in the ALFA+ cohort mainly come from a homogeneous social background, and we found no significant differences in household income or occupation depending on the number of biological children (eTable 7). Therefore, future research on the effect of pregnancy on women's brain health should disentangle the biological vs social aspects of this phenomenon by using a more diverse population with higher variability of social backgrounds.

Some limitations should be considered. First, time between visits is short, meaning that the reduced window of observation in our data probably limits the size of the effect observed. The number of follow-up visits is also insufficient to observe nonlinear evolution, thereby limiting our understanding of plausible mechanisms underlying AD pathogenesis. Future work should include additional time points to understand the trajectory of these results better. Second, although our sample's excellent health record makes it ideal to identify subtle changes in the early AD continuum, most ALFA+ study participants are Caucasians, resulting in an ethnically homogeneous sample primarily from middle-class to upper-middle-class backgrounds. In addition, excluding pregnancies affected by preeclampsia or gestational diabetes, although it strengthens internal validity by minimizing confounding from known vascular and metabolic risk pathways, may limit cross-study comparability. Future work should reproduce these analyses in a more diverse cohort to understand the effect of ethnicity and social background on the present results. Third, our sample size may have prevented the detection of smaller effects, especially regarding interaction terms. The very low number of grand multiparous women, in particular, limits the interpretability of our results, since parity could show a nonlinear relationship with the tested outcomes. Our findings represent preliminary evidence in an understudied area due to the lack of information relating to reproductive history in most large-scale AD cohorts. Replication in larger samples is therefore needed. In addition, the ALFA+ cohort is highly enriched for the AD genetic phenotype,^44^ with nearly 40% of participants carrying *APOE*-ε4 and many being offspring of patients with AD. This is not representative of the general population's genetic or familial risk profile and could have influenced our results. Finally, the absence of direct hormonal measurements, or measure of hormonal exposure, limits our ability to draw definitive conclusions about the role of sex steroid hormones in the observed associations. By relying on proxy measures, we can only estimate hormone-related effects, potentially leading to an underestimation of their actual influence.^45^

To conclude, this research showed preliminary evidence that parity is associated with postmenopausal women's cognitive trajectory and HV, depending on their Aβ status. Although parity did not directly influence cognitive performance, it interacted with Aβ status to shape both cognitive changes and HV. By highlighting how reproductive history interacts with Aβ pathology, our results may help reconcile previously inconsistent findings on the association between parity and AD risk and underscore the importance of considering female-specific biological factors in AD research. In summary, this work contributes to a more nuanced understanding of why women exhibit more deleterious downstream effects of AD pathology and highlights the importance of incorporating sex-specific and gender-specific risk factors in prevention and early AD detection strategies.

## Appendix Coinvestigators

**Table:**

Coinvestigators are listed in the Supplementary Material at Neurology.org/N.

## Glossary

Aβ: β-amyloid
AD: Alzheimer disease
CU: cognitively unimpaired
HV: hippocampal volume
MCI: mild cognitive impairment
mPACC: modified Preclinical Alzheimer's Cognitive Composite
TIV: total intracranial volume
